# Fluorescent, Bioluminescent, and Optogenetic Approaches to Study Excitable Physiology in the Single Cardiomyocyte

**DOI:** 10.3390/cells7060051

**Published:** 2018-05-31

**Authors:** Connor N. Broyles, Paul Robinson, Matthew J. Daniels

**Affiliations:** 1 Division of Cardiovascular Medicine, Radcliffe Department of Medicine, University of Oxford, Oxford OX3 9DU, UK; Connor.Broyles@rdm.ox.ac.uk (C.N.B.); paulr@well.ox.ac.uk (P.R.); 2 BHF Centre of Research Excellence, University of Oxford, Oxford OX3 9DU, UK; 3 Department of Cardiology, Oxford University NHS Hospitals Trust, Oxford OX3 9DU, UK; 4 BHF Centre of Regenerative Medicine, University of Oxford, Oxford OX3 9DU, UK; 5 Department of Biotechnology, Graduate School of Engineering, Osaka University, Mihogaoka 8-1, Ibaraki, 567-0047 Osaka, Japan

**Keywords:** calcium, voltage, fluorescent protein, bioluminescence, optogenetics, chemical dye, cardiomyocyte, FRET, adult ventricular cardiomyocyte, iPS-cardiomyocyte, excitable cell physiology

## Abstract

This review briefly summarizes the single cell application of classical chemical dyes used to visualize cardiomyocyte physiology and their undesirable toxicities which have the potential to confound experimental observations. We will discuss, in detail, the more recent iterative development of fluorescent and bioluminescent protein-based indicators and their emerging application to cardiomyocytes. We will discuss the integration of optical control strategies (optogenetics) to augment the standard imaging approach. This will be done in the context of potential applications, and barriers, of these technologies to disease modelling, drug toxicity, and drug discovery efforts at the single-cell scale.

## 1. Introduction

The human heart contains billions of cardiomyocytes generating the repetitive force required to pump blood around the body for the lifetime of the animal. Each cardiomyocyte (typically 100 μm × 20 μm × 20 μm in size) contains contractile units, called sarcomeres. These are arranged with ~2 μm spacing, contracting and relaxing 10% of that distance during each cardiac cycle. Hence, the only way to deliver a meaningful cardiac output is to synchronize contraction across many sarcomeres in a single cell, and many cells in a single heart. Failure to generate, or coordinate, cardiac contraction is a hallmark of inherited and acquired disease states. Therefore, understanding this process from the single cell to whole organ scale has been a driving motivational factor for cardiovascular research.

This review examines the development and application of tools to visualize how cardiomyocytes turn on, and off, in health and disease at the single-cell scale. We do not consider issues related to preparation, or isolation of single cardiomyocytes from neonatal-, adult-, or stem cell-derived sources, nor do we consider the historical backdrop acquired through the use of patch clamp electrophysiology that these methods have been calibrated against and are designed to complement or, in some cases, replace. Each topic in this review has its own literature so our aim is only to highlight tools which have proven or potential relevance to the single cardiomyocyte to provide a practical overview of how they work, and the known pitfalls in their application.

## 2. Chemical Dyes

Ionic movement in skeletal muscle cells was first recorded by Ridgway and Ashley in 1967 [1], using the naturally abundant jellyfish protein aequorin to measure intracellular calcium (Ca^2+^). In the pre-genomic era the method was hampered by the need to isolate and exchange the protein into chemically-permeabilized muscle cells. While this facilitated protein and ionic exchange, it prevented studies in intact cells, and required experiments to be conducted where jellyfish supply was plentiful. This motivated effort to recapitulate the properties of aequorin without these limitations.

### 2.1. Calcium Dyes

Measurement of intracellular Ca^2+^ in living cells was revolutionized by Roger Tsien in 1980 [2], when he reported fluorescent derivatives of the Ca^2+^ chelator 1,2-bis(o-aminophenoxy)ethane-*N,N,N′,N′*-tetraacetic acid (BAPTA). The properties of commonly used Ca^2+^ dyes are shown in Table 1. These were the first small molecules to reversibly and, specifically, bind Ca^2+^ with little sensitivity to other cations, like magnesium (Mg^2+^) and protons (H^+^). Initially they were impermeable and delivery required microinjection. Formulation as an acetoxymethyl (AM) ester overcame this and indicators like quin2 [3] were produced and rapidly applied to cardiomyocytes [4,5]. Quin2-AM can cross the plasma membrane and accumulate in isolated cardiomyocytes after brief incubation allowing Ca^2+^ transient recording. Calibration was achieved by selective cell permeabilization using either triton (to disrupt all cell membranes) or digitonin (to selectively disrupt the plasma membrane) [3]. Initial reports [4,5] identified similar basal (diastolic) Ca^2+^ levels (137 nM and 181 nM, respectively). However, the authors also noted technical concerns of chemical dye use: firstly, formaldehyde, acetic acid, and proton production by AM ester hydrolysis; and, secondly, attenuated contractility attributed to Ca^2+^ buffering as similar effects can be shown with ethylene glycol-bis(β-aminoethyl ether)-*N,N,N',N'*-tetraacetic acid (EGTA).

Quin-2 has single excitation and emission wavelengths; and thus is intensiometric. Therefore, overall fluorescence intensity changes can be affected by indicator concentration, a parameter that is difficult to control due to the passive diffusion mechanism of intracellular accumulation. Robust use of quin2 proved technically difficult due to its poor dynamic range, brightness, and inaccurate calibration. The second generation of intensiometric chemical Ca^2+^ dyes, such as fluo-3 [6,10] and fluo-4 [7,11], were ~30 times brighter than quin-2, so lower dye concentrations are required for dynamic imaging in cardiomyocytes. Improved brightness allowed a change in the imaging strategy from high sensitivity light collecting photomultiplier tubes (PMT) to high-speed video recordings which capture spatial detail.

This generation of dyes also introduced ratiometric tools, such as indo-1 [9,12] and fura2 [9,13], which have either single excitation, dual emission (indo-1) or dual excitation, single emission (fura2) fluorescent properties. Ca^2+^ binding to these sensors causes a change in one parameter whilst the second remains constant, providing an internal calibration of the indicator and allowing [Ca^2+^] to be determined independently of variability in indicator loading. Measurement of real-time ratiometric fluorescence requires bespoke imaging infrastructure for fura-2 or indo-1 due to the timescales dictated by the cardiomyocyte.

#### 2.1.1. Calibration

Initial calibration of the ratiometric indicator indo-1 used the in vitro dissociation constant (K_d_) to estimate intracellular Ca^2+^ by:

$${\left[{\mathrm{Ca}}^{2+}\right]}_{i}=\mathrm{Kd}\cdot \beta \left[\frac{\left(R-R_{\min}\right)}{\left(R_{\max}-R\right)}\right]$$

where β = 3 (the ratio of the free/bound Indo-l fluorescence at 485 nm), Kd = 250 nM, and R is the fluorescence intensity (F) emission ratio at 405 nm and 485 nm (F_405_/F_485_).

Followed by manipulation of intracellular Ca^2+^ using stepwise increases in membrane potential via a patch pipette [12], subsequent studies have shown a two to eight-fold discrepancy between the in vitro K_d_ of many Ca^2+^ indicators and the apparent Ca^2+^ binding affinity once loaded into living cells [14], questioning the validity of these calibration efforts. For example, the K_d_ of one of the more consistent dyes, Fluo-3, in vitro is 390 nM, versus 810 nM in the cytoplasm, or 620 nM in the nucleus. By contrast K_d_ for Calcium Orange is 185 nM in vitro, 1100 nM in the cytoplasm, and 1600 nM in the nucleus.

Complex protocols to calibrate fura2 use caffeine to open ryanodine receptors and empty the sarcoplasmic reticulum (SR) Ca^2+^ store, with thapsigargin to block sarco/endoplasmic reticulum Ca^2+^-ATPase (SERCA)-mediated Ca^2+^ reuptake. Subsequently selective plasma membrane permeabilization using a Ca^2+^ ionophore (e.g., ionomycin) and 2,3-butanedione monoxime (to inhibit myocyte contraction) allows a titration curve with defined free Ca^2+^ solutions to plot [Ca^2+^] versus the fluorescence ratio [15,16,17]. This procedure is technically challenging and frequently adds layers of error onto the raw fluorescent data. As a result, most publications, which do not require the absolute determination of intracellular [Ca^2+^], present a raw ratio.

**Figure 1:**
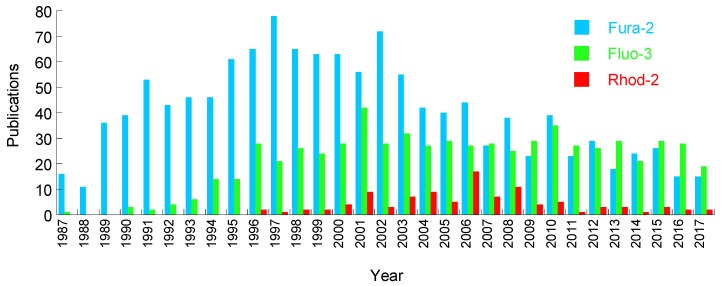
Annual publications imaging cardiomyocytes or heart tissue using the fluorescent Ca^2+^ indicators fura-2, fluo-4, and Rhod-2. Metrics extracted from CSV data at Pubmed.

After the initial wave of calcium indicator development, many groups have subsequently evolved the core BAPTA chemistry to generate a spectrum of indicators with a range of Ca^2+^ sensitivities. Their properties and uses have been previously reviewed [18]. Increasingly detailed experimental applications to study the mechanisms that underlie excitation:contraction coupling paralleled probe development, delivering improved accuracy and fidelity throughout the 1990s and 2000s (Figure 1). Optical improvements, and subsequent commercialization of bespoke imaging platforms (e.g., from IonOptix and Aroura), focused on measuring dye-based cardiomyocyte Ca^2+^ and contractility have supported the broader uptake of these tools.

Advanced applications, for example, the measurement of free and myofilament bound Ca^2+^ in intact cardiomyocytes, can be achieved using chemical Ca^2+^ indicators in combination with a simultaneous voltage clamp. The first step of these protocols requires intracellular Ca^2+^ to be revealed either by a calibrated fura2 transient or the intensiometric signal of fluo4, which can be calibrated by mechanical disruption of the plasma membrane at the end of each experiment to obtain the maximum fluorescence (F_max_) relative to the external Ca^2+^ concentration. In the second step, estimates of free Ca^2+^ are plotted against total intracellular Ca^2+^, determined by the calculation of the Sodium Calcium Exchanger (NCX) integral conferred by changes in whole cell membrane potential upon the direct application of caffeine, where the difference between free and total Ca^2+^ reflects myofilament-bound Ca^2+^ [17,19,20].

#### 2.1.2. Subcellular Applications

Dyes typically occupy the entire cell volume, yet high-speed confocal imaging by line scanning, in conjunction with brighter indicators (e.g., fluo-3) reveals subdomain phenomena, such as Ca^2+^ sparks. Ca^2+^ sparks represent individual Ca^2+^ induced Ca^2+^ release (CICR) events from the SR via the ryanodine receptor (RyR) at the dyadic cleft of the transverse tubule (T-tubule) [21]. Changes in spark frequency are thought to be indicative of RyR leak, an important hallmark of heart failure and cardiac disease. However, Ca^2+^ spark events are short lived, therefore, adequate sampling for reliable spark frequency quantitation needs line scanning speeds over 500 Hz (~2 ms/line) [22]. Imaging at this speed requires high-sensitivity detectors so that illuminating laser power intensities can be reduced, minimizing photobleaching and ensuring maximal spark detection. Ca^2+^ spark studies can be combined with chemical voltage dyes to study beat-to-beat T-tubule specific events in response to small molecules [23] or disease-causing mutation [24].

Organelle-level information can be derived from indicators with affinities that work in high [Ca^2+^] environments. Since they saturate in lower [Ca^2+^] conditions, the dynamic signal is presumed to arise from the more concentrated environment. One example of this is the low-affinity intensiometric green indicator Fluo-5-N, which has been used in studies to detect Ca^2+^ within the cardiac SR [8] where Ca^2+^ concentration is estimated to be 0.5–1 mM (>1000× the cytoplasm). To achieve localized fluorescence requires a change to the typical 10 min loading protocol. Prolonged dye incubation (2 h) and extended washout (1.5 h) to remove cytoplasmic Fluo-5-N AM is needed. This approach reveals beat-to-beat fluorescence intensity reductions, with a T-tubular/SR distribution, as the SR empties and refills during the contractile cycle. As the indicator for SR Ca^2+^ is intensiometric, calibration was determined by estimating F_max_ firstly by incubating intact cardiomyocytes with isoproterenol, then adding tetracaine to block SR Ca^2+^ leakage and, finally, removing sodium (Na^+^) to raise both intracellular and SR Ca^2+^. Cells were then permeabilized with saponin and caffeine added to allow Ca^2+^ equilibration across the SR. Finally, cells were incubated with variable free Ca^2+^ solutions in the presence of ruthenium red, oligomycin, and cyclosporine to limit mitochondrial Ca^2+^ uptake.

Attempts have also been made to measure mitochondrial Ca^2+^ dynamics in the cardiomyocyte using the red indicator Rhod-2 [6,25,26], which partially localizes to the mitochondrial network (~60%) due to its positive charge. As residual cytosolic localization of Rhod-2 persists, several deconvolution experiments are necessary to filter mitochondrial-specific Ca^2+^-dependent fluorescence from the total signal. This relies on the observation that the upstroke of mitochondrial Ca^2+^ slightly precedes the upstroke of cytoplasmic Ca^2+^ upon electrical stimulation of the cardiomyocyte with a patch pipette. The temporal difference is only observed after CICR from the SR is forced by modulation of extracellular Ca^2+^ and Na^+^ and compared to peak cytoplasmic Ca^2+^ fluorescence timings for each condition. Finally, detailed patch clamping efforts were needed to cross-check findings in order to establish the intrinsic latencies of two known mitochondrial voltage-gated Ca^2+^ channels (mCa1 and mCa2) [27]. The authors were unable to calibrate Ca^2+^ levels in the mitochondria due to the intensiometric nature of Rhod-2.

At the other end of the size scale fura2 has been loaded into whole Langendorff perfused mouse hearts. Surface mapping of fluorescence ratio has been used to measure Ca^2+^ in the whole heart [28]. This required development of low-magnification, high-sensitivity optics with fast switching filter sets to measure the ratiometric fluorescence at the macroscopic scale.

#### 2.1.3. Disadvantages of the Calcium Dyes

This section has highlighted the positive aspects of chemical Ca^2+^ indicators as tools to measure intracellular Ca^2+^. Yet it is clear that even with careful calibration and cross-checking with voltage clamp data, there is limited information on subcellular Ca^2+^ domains with these tools. Furthermore, it should be noted that the complex calibration protocols that underpin quantitative efforts have rarely proliferated beyond the groups that initially establish them. This may be due to the difficulties maintaining bespoke optical systems in combination with high fidelity patch clamp setups and the technical expertise to run them.

The metabolites of the AM-ester, and the requirement for indicator loading in the presence of the nonionic surfactant polyol Pluronic F-127, which improves the solubilization of the otherwise water-insoluble dye, introduce additional cofactors that may change the native physiological function of the cardiomyocyte.

Furthermore, it appears that Ca^2+^ dyes might do more than just indicate Ca^2+^. For example, BAPTA-derived Ca^2+^ indicators affect at least two key ATPases in the cardiomyocyte. Firstly, Na/K ATPase inhibition occurs in many cell types, limiting cell survival and possibly reducing ATP-dependent force production [29]. In addition, we recently found that fura-2, and a number of chemically-unrelated dyes, reduce contractility in both adult guinea pig ventricular cardiomyocytes (vGPCM) and induced pluripotent stem cell (iPSc)-derived cardiomyocytes, similar to observations first described in the initial quin-2 studies over 34 years earlier. Contractile impairment by fura-2 is driven by reduction in the Ca^2+^ sensitivity and also maximal rate of actomyosin ATPase activity [30]. Therefore, it appears that chemical Ca^2+^ indicators may directly inhibit actomyosin ATPase itself, raising the possibility that, in addition to the Na/K ATPase, other ATPases may also be vulnerable to chemical dyes.

### 2.2. Non-Calcium Sensitive Dyes

#### 2.2.1. Sodium Indicators

Chemical indicators have been developed for more than Ca^2+^; given the central importance of depolarization provoking CICR, intracellular Na^+^ indicators, such as the sodium-binding benzofuran isophthalate (SBFI) (Table 2), have been developed [31]. The application of SBFI to the heart has been modest compared to Ca^2+^ indicators, however, 70+ publications have used this tool in cardiomyocytes (source: NCBI/PubMed). To date, applications have been restricted to the calibration of basal Na^+^ levels in the heart, as beat-to-beat Na+ transients are too fast and the dye’s brightness and dynamic range are inadequate for depolarization studies [32]. Initial attempts to calibrate Na^+^ levels noted that 50% of the SBFI fluorescence was retained upon chemical permeabilization with either saponin or digitonin. Residual fluorescence was sensitive to cyanide or carbonylcyanide m-chlorphenylhydrazone (CCCP), suggesting mitochondrial uptake may occur [33]. To date, no reports of subdomain sodium analyses in the cardiomyocyte using a chemical dye exist. These measurements have been driven by voltage clamp studies and fall beyond the scope of this article.

#### 2.2.2. Voltage Dyes

Membrane potential indicators, such as the hemicyanine class of electrochromic indicators, like di-4ANNEPS [34], and photo-induced electron transfer-based probes, like FluoVolt [35], are more widely used to study depolarization. The properties of commonly used tools are shown in Table 2. In contrast to the chemical Ca^2+^ dyes, which preferably work within the cytoplasm of the cell, membrane potential indicators have hydrophobic (butylated) tails to facilitate localization to the plasma membrane.

Sulphydryl-based indicators, like di-4ANEPPS, which rely on the rapid Stark effect for their speed, can temporally report membrane depolarization in isolated cardiomyocytes [38], or optically map the ventricular wall surface to report the whole heart action potential (AP) [39]. A broad spectral palette of hemicyanine dyes have been developed by fluorination and slight modification of the di-4ANNEPS prototype [36] with emissions extending from yellow (Di-4ANE(CF3)EPTEA (Ex 418 nm/Em 597 nm) to the near-infrared (Di2-ANBDQ(F)TEA) (Em 553 nm/734 nm). These dyes retain the small hypso- or bathochromic wavelength shifts in absorption and emission spectra in response to the electric field, and have improved photo-stability, thereby extending the experimental timescale. The far red dye Di-4-AN(F)EPPTEA has been used to assess whole heart membrane potential [40] and in dual-color single-cell studies. One particular advantage of the hemicyanine dyes is the large Stokes shift between absorbance and emission spectra (typically ~170 nm). This makes it possible to combine these tools with other probes for single-excitation, dual-emission experiments of Ca^2+^ and voltage [23,24]. However the signal: noise of these probes is often limited (typically ~10%/100 mV), and their fluorescence response to voltage change is strongly excitation wavelength dependent (e.g. 100% for di-4-ANNEPS [35]).

Although generally disadvantageous, this phenomenon can be exploited by an approach that appears to boost overall signal:noise called shifted excitation and emission ratioing (SEER). SEER requires two wavelength excitations, and two wavelength emission detections. Since both the excitation and emission spectra are voltage sensitive, the ratio of the shifted emission intensity triggered by the shifted excitation to the initial emission intensity triggered by the initial emission reveals the maximal difference provoked by depolarization. Indeed, the final image processing step ultimately appears to double the measured signal from commonly used dyes, like di-8-ANEPPS [41]. Finally, hemicyanine dyes operating in the near-infrared have been described [37], which avoid the auto-fluorescent emissions of hemoglobin and are compatible with optogenetic stimulation protocols using Channelrhodopsin [42] with minimal spectral interference.

As signal strength is a limiting factor with hemicyanine dyes, a Förster energy resonance transfer (FRET) based approach to voltage imaging using a dye-only approach [43] or a hybrid genetic probe:dye strategy [44] was developed. These methods utilize the steep spatial dependence of FRET (<10 nm) to be able to detect the redistribution of membrane-integrated anions in response to electric charge. When the light donor, and acceptor are on the same side of the membrane FRET can occur, this signal is broken when one component preferentially redistributes in response to voltage. Signals with this method are typically larger than electrochromic dyes [35], but the strategy introduces capacitance into the cell membrane. The process of dye flipping between intra- and extra-cellular membranes is relatively slow. This may prove limiting in some neurobiology applications where activations can occur up to 100 Hz, but cardiac cells are less demanding (0.5–5 Hz) and this aspect may be less relevant.

The final dye-based class of voltage sensors use photon-induced electron transfer to emulate the best parts of both of the previous strategies. FluoVolt incorporates fluorescently-active 2,7-dichloro-6-hydroxy-3-oxo-3H-xanthene (analogous to the fluorescent element in Fluo-3) giving a brighter signal, which is faster and less prone to bleaching in response to membrane depolarization. It is retained in the cell membrane longer than di4-ANEPPS, thereby increasing experimental duration. Cell loading of FluoVolt requires AM ester dispersal with Pluronic F127 and can yield toxic formaldehydes, as for the Ca^2+^ indicators detailed above. Therefore, concerns remain regarding suitability when assessing native cardiomyocyte function; for example, single cell contractility is reduced in the presence of FluoVolt [30], a phenomenon documented in previous studies using the hemicyanine dye RH421 [45]. To date, we have identified only two studies exploring cardiac application of FluoVolt; both utilizing iPSc cardiomyocytes in drug screening studies [46,47].

In summary, VSDs are more widely used to study deplolarization events in single cardiomyocytes than analyte-based measurements of sodium. The most commonly used dyes are the hemicyanine class, which have many spectral variants and long Stokes shifts. Cardiomyocyte contractility may be impaired by at least some of the indicators in this group [45]. Although improvements have been made to their performance [36], signal to noise is often limiting, and experimental durations are brief. FRET-based approaches improve on brightness, but relatively lack speed and increase membrane capacitance. Photo-induced electron transfer probes are the most recently developed class [35], although they have improved wavelength-independent voltage sensitivity, retain rapid kinetics, and have minimal impact on capacitance their signal in cell models is similar to the best-performing hemicyanine dyes and, in isolated cells, contractile impairment remains detectable [30].

#### 2.2.3. Others

Despite continued concerns about their suitability to measure ionic movement in the native cardiovascular system and, in particular, their ability to do that without undesirable off-target effects, fluorescent chemical dyes have become commonplace since their initial discovery over the last 30 years (Figure 1). In fact, many subtypes of cation indicator have been produced to detect zinc (FluoZin-3 or RhodZin-3) [48], Mg^2+^ (Furaptra [49], Mag-fura2 [50] and Mag-Indo1 [51]), and H^+^ (SNARF-1) [52], although detailed off-target studies have not been conducted in cardiomyocytes at this point.

## 3. Genetically-Encoded Sensors

**Figure 2:**
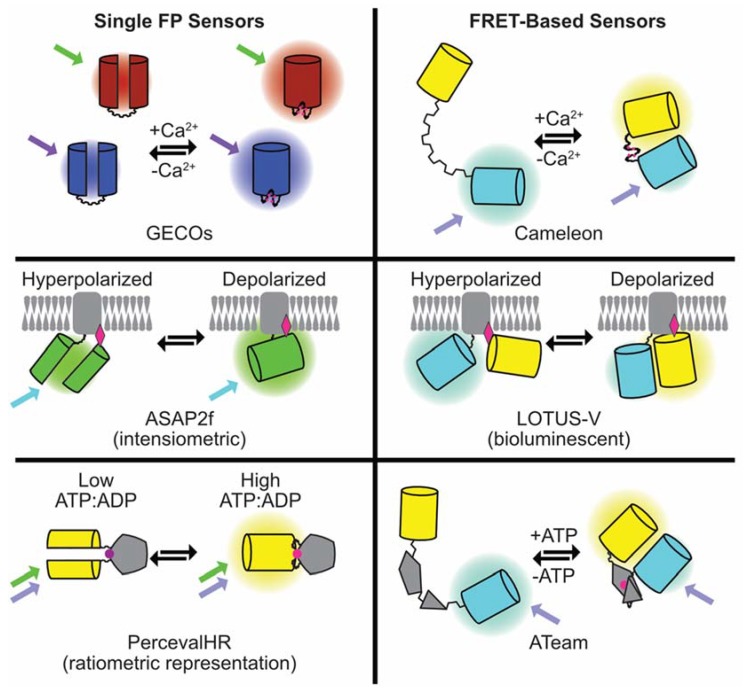
Summary of the composition and mode of action of the genetically-encoded fluorescent sensors for Ca^2+^, voltage, and ATP discussed in the article.

Although the periodic light-emitting properties of aequorin in isolated muscle cells [53] spurred the development of chemical dyes at a time when tools to manipulate and deliver genetic material were lacking, it has always been possible to imagine that proteins may offer an alternative method of illuminating cellular physiology. Theoretical advantages of genetically-encoded indicators include restriction to certain cell types, or subcellular compartments, by the use of specific promoters or defined tags. As genuine subcellular probes complex, calibration experiments should be unnecessary provided that localization can be unambiguously defined. Unlike chemicals, which continuously accumulate to mM levels, steady-state protein concentrations are often in the μM range. Although this makes them less toxic, it also makes them harder to see, particularly when the light emission is at the low levels typical of aequorin. Brighter fluorescent probes for Ca^2+^, voltage, and second messengers and metabolites, such as cyclic adenosine monophosphate (cAMP) and adenosine tri/di-phosphate (ATP/ADP), have now been made and applied to cardiomyocytes. Sensor domains can be inserted into single, or between, fluorescent protein (FP) pairs to produce intensiometric or ratiometric indicators analogous to the chemical dye strategies described previously. An overview of genetically-encoded indicator types discussed in this article is shown in Figure 2.

Ratiometric indicators commonly rely on FRET—a phenomenon that exists when specific FP and/or dye color combinations are appropriately orientated and distanced from one another, allowing non-radiative transfer of energy by dipole-dipole coupling. Alteration in the sensor domain structure, by ligand binding, changes the spacing and/or the angle between the donor and acceptor chromophores producing a variable signal that can be measured. In its simplest form a FRET-based ratiometric measurement needs a donor and an acceptor image. As FRET increases, the acceptor will get brighter (sensitized emission), while the donor will get darker (donor quenching). Therefore, this technique relies on relative shifts of light detected rather than the absolute emitted light intensity, making it quantitative and independent of the expression level and movement. However, on the millisecond timescales needed to observe a single cardiomyocyte contraction it can be difficult to acquire two perfectly-aligned images rapidly without a beam-splitter, and/or dual-view cameras. Similarly, the presence of two FPs makes these probes bulky, physically, and optically, as the effective FRET pairs available currently tend to cover ~150–200 nm of the visible spectrum. This makes it difficult (but not impossible [54]) to view combinations of ratiometric tools in single cells. Single FP designs of ratiometric indicators [55,56] use conformational change induced by the substrate binding to promote rearrangement of the chromophore and alter the emission wavelength. Their smaller size and optical simplicity, therefore, offers significant advantages over FRET-based tools.

By contrast, intensiometric indicators based on single FPs get brighter/darker in response to substrate binding/sensing as the physical environment around the chromophore changes, regulating the photon number. Intensiometric probes are small and simple to use because they require single wavelengths for excitation and emission. They also tend to have larger dynamic ranges. Collectively these attributes have made them more popular than their ratiometric cousins. However, because they are based on brightness alone, results can be affected by the expression level, or even subtle changes that occur to the number of molecules in a region of interest as the cardiomyocyte contracts, creating a motion artefact.

### 3.1. Genetically-Encoded Calcium Indicators 

The most widely used and best performing group of protein indicator tools measure Ca^2+^. The ideal genetically-encoded calcium indicator (GECI) for cardiomyocytes would be easy to use, physically small, bright when Ca^2+^-bound, fast, ratiometric, have affinities tuned to enable calcium observation in various subcellular locations, and be available in multiple colors for simultaneous observations. They should not disturb the natural behavior of the cell even though they will inevitably buffer intracellular Ca^2+^.

The first fluorescent GECI was developed following the discovery of green fluorescent protein (GFP) and the production of the blue and yellow color variants with the required (ideally >30%) spectral overlap FRET demands. The original GECI, Cameleon [57], sandwiches a Ca^2+^ binding domain next to a Calmodulin (CaM) kinase domain between the cyan (CFP) donor and yellow (YFP) acceptor FP pair. The indicator has a K_d_ of 5.4 μM, with an 80% signal change when Ca^2+^-saturated. Since the beat-to-beat Ca^2+^ flux of the cardiomyocyte is between ~0.25 and ~1 μM, it was not possible to visualize single cardiomyocyte transients with this tool until its iteration YC2.1 (K_d_ 0.1 μM, 100% signal change) was reported and tested in neonatal rat cardiomyocytes [58]. Although this gave a durable signal for several minutes in this model, YC2.1 was quickly overtaken by parallel developments to produce single FP GECIs.

When successful circular permutation (cp) of GFP was identified, it became possible to develop GECIs based on single FPs [59]. In this model the halves of the FP are reconstituted when the ligand is present, and the probe therefore alternates between a dim, and a bright state. Not only does this strategy produce small indicators from single FPs, it also significantly improves their dynamic range as the background signal in the Ca^2+^ free state is very low. Various families of single FP GECIs are now described, the comprehensive analysis of Kaestner et al. [60] highlights the progress made in single cardiomyocyte applications up to 2014, principally with the Pericam (derived from the YFP variant Venus) [61] and the early GCaMP series (derived from GFP) [62] of indicators.

A few observations from [60] are worth repeating, as they are likely to be generalizable to the other types of protein-based indicators:

(1) In head-to-head comparisons between GCaMP2, and Fura2, in the same cells, at the same time, the dye appears faster (on and off). Subsequent iterations have improved the speed of the GECI response.
(2) When compared to Fura2, GCaMP3 produced a comparable signal to noise ratio, but with a Ca^2+^ buffering capacity at least an order of magnitude smaller than the dye.
(3) The ratiometric indicator TN-XXL (K_d_ 0.8 μM, 136.5% signal change) [63] and the intensiometric indicator Flash Pericam (K_d_ 0.7 μM, 700% signal change) [61] were non-functional in adult cardiomyocytes, even though cells were successfully infected and in vitro performance suggested appropriate K_d_ and dynamic range. Therefore, it cannot be assumed that all potentially appropriate indicators will work in all cardiac model systems until they are tested. Since dyes change their K_d_ in the cellular environment [14] it is possible that protein-based indicators face similar challenges. This may make reliable and accurate calibration extremely difficult.
(4) Although the reported dissociation rates for the GECIs in vitro at room temperature can be close to one second, in cellular preparations the observed decay (*τ_Off_*) rates are adequate to see signals return to baseline with all indicators tested.
(5) Adenovirally-delivered YC3.6 in a rat adult ventricular cardiomyocyte model at 48 h had an order of magnitude difference in the expression level (~0.4–4 μM), which corresponded to a similar variation in observed emission intensity.

In summary, up to 2014, a number of GECI strategies had been developed and, on a few occasions, tested in cardiomyocyte models. Rarely, subcellular calcium stores (e.g. the SR [58] or the mitochondria with an inverse Pericam [64]) were probed in single cardiomyocytes with these tools. The perceived weaknesses were speed (relatively slow), dynamic range compression (in vitro this parameter can exceed 700%, but in cells typically 30–40% is seen), and a lack of spectral variants (everything is in the blue:green:yellow end of the spectrum).

#### 3.1.1. Extending the Palette of GECIs; the GECOs

From 2011 the scope and the scale of the screening efforts to produce GECI color variants, with greater intensity changes and faster kinetics increased. This led to the production of the genetically-encoded calcium indicators for optical imaging (GECO) family of blue, green, and red indicators (B-GECO, G-GECO, and R-GECO), with spectral compatibility allowing observation of calcium in multiple locations within a single cell [55]. Further expansion of the color palette of GECOs included the production of the furthest red, CAR-GECO1 (peak emission 609 nm, K_d_ 490 nM, signal change 2700%), and the greatest signal change from an orange, O-GECO1 (peak emission 565 nm, K_d_ 1.5 μM, signal change 14,600%) variant [56,65]. Ratiometric GECOs were simultaneously produced from either two FP components, the blue/green GEM-GECO [55], or even from single FPs with ratiometric properties derived from the isosbestic point of the excitation spectrum, to produce green GEX-GECO [55], and subsequently red REX-GECO [65]. The ratiometric single FP probes should be resistant to motion artefacts, and retain spectral bandwidth for other probes or optogenetic tools. However, these ratiometric indicators have relatively low dynamic ranges, slow kinetic parameters, and a limited range of Ca^2+^ affinity and are currently unproven in cardiomyocytes.

#### 3.1.2. Affinity GECO Variants

A range of Ca^2+^ affinity GECI’s have been developed particularly for the red indicators. For example, R-GECO1, (K_d_ 0.223 μM, range 870%) was tuned to a less sensitive form R-GECO1.2 [55] with a K_d_ = 1.2 μM, and subsequently the development of lower affinity red GECOs (LAR-GECOs) for high Ca^2+^ environments with K_d_ of 24 μM and 12 μM for LAR-GECO1 and 1.2, respectively [66]. The G-GECO, R-GECO, and GEM-GECO template were also used in the CEPIA series of low-affinity indicators G-CEPIAer (K_d_ 672 μM, range 470%), R-CEPIAer (K_d_ 565 μM, range 880%), and GEM-CEPIAer (K_d_ 558 μM, range 2170%) [67], which have Ca^2+^ affinities three orders of magnitude lower than the parental compounds.

To date successful application of the GECO series in single cardiomyocytes has been reported [30,68,69]. Little investigation has been conducted in phenotyping subcellular compartments in the cardiomyocyte [30], but the tools available could in principle be used for this purpose.

### 3.2. GCaMP Series

The GCaMP series of indicators are circularly permuted GFPs containing a calcium-sensing domain (M13/CaM), and by the volume of publications are the more commonly used tool. The initial empirical and then structure-guided evolution produced the breakthrough GCaMP3 (K_d_ 345 nM, range 1350%), which was functional in transgenic models [70]. The next notable evolutionary step of the GCaMP series turned strongly in the neurobiology direction in 2013 when the Janelia Farm group reported the development of a screening platform based on cultured neuronal cells [71]. This represents a step change away from the bacterial paradigm used for the Cameleon, Pericam, and GECO probes towards the cells they will ultimately be used in. In this landmark effort successful testing of 447 variants identified the green GECI GCaMP6f (K_d_ 375 nM range 5180%), which boasts the greatest signal change and faster on/off kinetics compared to existing probes [71]. Unfortunately, because several groups have taken on the task of improving GCaMPs, the nomenclature has the potential to get (and to our minds has become) confusing. For example, a GCaMP6 (with mutations N105Y, E124V, M36L, D78Y relative to GCaMP3) has been reported by the original GCaMP inventors [72] and retains the original nomenclature for the amino-acid changes while, in the parallel efforts of the Genetically-Encoded Neuronal Indicator and Effector (GENIE) consortium [71], a different G-CaMP6 and numbering system based on the raw sequence rather than its historical context has been chosen (for example their GCaMP6f is T302L, R303P, A317E, D380Y, T381R, S383T, and R392G relative to GCaMP3). Although there are many comparative studies of the various indicator series in neuronal models, comparative studies in cardiomyocytes of the GCaMP indicators are lacking and, therefore, at this point, it is not possible to assert which of the tools is preferred in which model system.

Interestingly, experimental data suggests this is a problem. For example, when used in the regenerative context, a transgenic GCaMP3 knock-in to the AAVS1 locus that worked in human embryonic stem cell (ESC)-derived cardiomyocytes delivered to the guinea pig [73] and the pigtail Macaque [74] did not produce a dynamic calcium transient in a macaque iPSc cardiomyocyte cardiac graft, and a separate probe was developed to overcome this (GCaMP7.09, M153K, T203V, N205S, N105Y, E124V, L36M, N60D, D78Y compared to the original GCaMP, K_d_ 212 nM, range 1900%) [75].

GCaMP6f has been incorporated into several platforms that show potential for high-throughput single cell assays. In 2015, Maddah et al. introduced Pulse, a video tracking system of contractility which includes a transcription activator-like effector nucleases (TALEN) targeted knock-in of GCaMP6f to the adeno-associated virus integration site 1 (AAVS1) locus [76]. Although most of the analyses presented used white light, GCaMP6f signals could be visualized in these cells. However, it is not clear whether the transients reported are from single cells, or aggregates, and in general terms the data presented suggest the signal intensity is low. A similar strategy was reported [77] with a different motion tracking algorithm, and although GCaMP6f signals are shown, they are from cell sheets rather than single cells. Therefore, based on these two studies it is possible the GCaMP6f might not offer the anticipated benefits in cardiomyocyte models that it does in neurons. GCaMP5G driven by the Troponin T promoter in a Lentiviral cassette does appear adequate in single stem cell cardiomyocytes and adult ventricular cells. It was used in single iPSc cardiomyocytes to observe the effects of small molecules and disease-causing mutations, including alongside the genetically-encoded voltage indicator ArcLight [78]. GCaMP5G is also a component of the simultaneous Ca^2+^ and voltage indicator system CAVIAR developed by the Cohen lab which has been tested in cultured and transgenic cardiomyocyte models [79,80]. GCaMP3, and GCaMP2 have been shown to be effective in isolated adult ventricular myocytes [60].

Combining GCaMP’s into multi-color experiments should be done with caution, as GCaMP3, 5, and 6 demonstrate photoconversion from green to red by exposure to the typical light sources used to image these probes (450–500 nm light). The red emission is stable, and remains Ca^2+^ responsive, but can be prevented if Valine115 (GCaMP3 nomenclature) is restored to threonine (as found in GCaMP2); photoconversion is enhanced in anaerobic environments [81].

### 3.3. RCAMPs

Red color variants, described as RCaMPs, have been developed but, again, the nomenclature is crowded and the reader is reminded that these are either derivatives of mRuby (an engineered variant of eqFP611, [82]) which was identified in the sea anemone *Entacmaea quadricolor* [83] or R-GECO, itself a variant of mApple, an engineered product of dsRed identified in the stony coral *Discosoma sp.* [84], rather than variants of the GFP from the North Pacific jellyfish *Aequorea victoria* that produced the GCaMPs. In practical terms this means that antibodies against GFP, which will work for its spectral variants (e.g., CFP, YFP), GCaMP, and B- or G-GECO derivatives will not work for any of the RCaMP families. Of the initial mRuby variants screened RCaMP1h (K_d_ 1.3 μM, range 1050%) appears preferable as it bleaches slower and irreversibly compared to its counterparts with decay kinetics about one quarter that of RGECO1 [83]. RCaMP1.07 (K_d_ similar to RGECO, range 2800%) in cultured neurons appears to produce a signal twice that of RGECO1, however, the mutations introduced potentially encode a nuclear export sequence, such that it is an exclusively cytoplasmic [84] feature also seen in the GCaMPs.

Iterations of the RCaMPs have been described which aim to narrow the performance gap with their green counterparts. RCaMP2 (K_d_ 69 nM range 480%) is a high-affinity, fast variant with relaxation rate constants 2–3 times that of RCaMP1.07, and a Hill coefficient that approaches that of the chemical dyes. As such, it can track single action potentials (AP) in cultured neurons up to 20–40 Hz [85]. By contrast, the Janelia team, with the high content neuronal culture screening platform [71], analyzed more than 1000 variants to identify jRCaMP1a and 1b from RCaMP1h, and jRGECO1a from RGECO1 [86], that appear to offer significant improvements on the parental protein. In the cultured neuronal model jRGECO1a is almost functionally equivalent to GCaMP6, but does accumulate in lysosomes. We have been unable to identify any literature on the application of the RCaMP tools to single cardiomyocytes, but they appear to have some advantages on paper that may be worth exploring.

#### Undesirable Properties of the Red GECIs

It should be noted that all red GECIs display a green emission (500–550 nm) that is not related to Ca^2+^, but which may potentially interfere with dual-color imaging studies. One major difficulty with the isolated adult cardiomyocyte is a lack of spontaneous activity and, thus, a requirement for pacing. Optogenetics (below) has been introduced as a more versatile and less toxic alternative to traditional electrical stimulation. However, while electrical pacing is simple to combine with the GECIs, their use with simultaneous optogenetics is more complex. Firstly, depolarizing optogenetic tools use blue-light for activation and, therefore, are incompatible with green GECIs that are activated at similar wavelengths. This forces the use of the red GECIs which generally underperform compared to the green probes. Successful combination of R-GECIs with optical control are reported for RGECO in single iPSc-derived cardiomyocytes for drug toxicity screening [68], and in neuronal models for various RCaMP combinations [60,81,83]. These studies show that red GECIs based on mApple have retained a light-induced photoactivation phenomenon that can make them get brighter even if Ca^2+^ is unchanged. This can be avoided by minimizing light exposure power, duration, and wavelength when indicators like RGECO and REXGECO are used, but does require care to differentiate artefacts from actual responses. The RCaMP series derived from mRuby appear less vulnerable to this problem [83,85]. A further class of red GECIs based on another *Entacmaea quadricolor* derivative, mKate, an FP that has negligible blue light activation, has given rise to the KGECOs which have equivalent performance to RGECO1 as a Ca^2+^ indicator and out-perform it during optogenetic stimulation in iPSc cardiomyocytes due to freedom from photoactivation [69]. This may make it preferable in high throughput single cell analyses which need to be conducted with as little supervision and experimental caveats as possible since any problems amplify with scale.

### 3.4. Bioluminescent Calcium Indicators

Bioluminescent Ca^2+^ indicators have been produced with improved brightness [87,88] but although these probes are suitable for low frame rate applications in single cells, their conversion to dynamic calcium indicators reduces their brightness and, to date, only GeNL has been demonstrated to be effective in small (cell n ≈ 100) clusters of iPS-CMs [88]. Affinity [88,89] and spectral [89] variants have now been produced. Nature’s original calcium indicator, aequorin, is bioluminescent rather than fluorescent. Thus, it is possible that this strategy may offer maximal biocompatibility, and from a screening perspective the best signal-to-noise. However, further improvements are likely required for application in single cardiomyocytes in order to meet the minimum 20 Hz imaging requirement to avoid temporal aliasing. Another major challenge is the gradual consumption of the chemiluminescent substrate over time, which gradually reduces brightness. 

### 3.5. Genetically-Encoded Voltage Indicators

Calcium sensors are a surrogate for the AP, but cellular voltage describes physiology better than one ion can. This may be preferable for drug or disease characterization [90]. While many genetically-encoded voltage indicators (GEVIs) have been produced, their performance is poor. In particular, the dynamic range is often 10%/100 mV or worse, with a brightness typically 100–1000× less than GFP. This makes them harder to work with, and more demanding on equipment infrastructure and environmental controls. As with the GECIs, the driving development needs are led by neurobiology. While cardiology does not require tools that are as quick, it will benefit from those that are brighter and ratiometric to mitigate motion artefacts arising during contraction. Contemporary reviews highlighting the most promising GEVIs in neurons are available [91].

GEVIs operate through a voltage sensing domain consisting of an entire, or a fragment of an integral membrane protein that undergoes structural change in response to voltage change (typically by protonation of a Schiff base) in order to reorganize the FP components causing a fluctuation in the FRET or fluorescent emission. Kaestner et al. summarized GEVI applications in cardiovascular research to 2015, highlighting the potential for GEVI use in cardiotoxicity screens with Mermaid [92,93] a green/orange ratiometric tool with a 48% signal change/100 mV in 293T cells. A proposed application was profiling iPSc cardiomyocytes based on their AP; which differs between the major cell-types in the heart. Current differentiation protocols produce a mixture of the cell types found in the heart. Therefore, identifying single cells as atrial or ventricular by their AP phenotype would reveal cell-specific effects without improving the differentiation process or requiring cell purification. This was explored using lentiviral delivery of ArcLight, a GEVI based on a pH sensing variant of GFP, with a 30% change/100 mV in single iPSc cardiomyocytes. Whereas the probe was typically ~60 ms slower than the simultaneous patched voltage, in-contrast to chemical dyes it extended observation windows from seconds to minutes in single cells [90]. As an intensiometric indicator, motion artefact was investigated and found not to impact the APD_90_ measurement in cell clusters [90]. Arclight was also used successfully to document the effects of small molecules and genetic mutation in single iPSc cardiomyocytes [78], including as a stable transgenic line. Kaestner et al. demonstrated successful application of VSFP-CR, a green-red GEVI based on mClover and mRuby (13%/100 mV) in the single iPSc cardiomyocyte model [93,94].

In contrast to the dramatic progress in improving the GECIs which went from 80% to ~800% signal changes in the first four years after they were developed, and to >10,000% changes within 15 years of their invention, making equivalent progress with the GEVIs using the same toolbox has proved impossible. In a change of strategy Inagaki et al. fused the bioluminescent light donor NanoLuc to the widely used voltage sensor domain from *Ciona intestinalis* used in Mermaid and the Venus YFP variant to produce LOTUS-V. Although bioluminescent tools are dimmer than fluorescent tools (which currently prevents their successful application to single cells) bioluminescent resonance energy transfer (BRET)-based GEVIs can be applied to iPSc cardiomyocyte clusters [95] and deliver a number of clear advantages, including better signal-to-noise, improved long-term recording (hours), independence from motion artefact (preserving validity of intensity measurements) and freedom from crosstalk with various optical control strategies that have a shared heritage with some of the GEVIs.

Of the GEVI improvements described to date the Arclight derivative (green) BongwooriR6 has improved on and off rates, and a shifted voltage sensitivity (V1/2 near 0 mV, rather than Arclight −20 mV) that makes it more suited to threshold depolarization events [96]. BongwooriR6 may not provide the speed of another green GEVI based on cpGFP called ASAP2f [97], but offers better signal strength. The red FlicR1 (3%/100 mV), a derivative of mApple, has faster kinetics that closely approximate the patched electrical trace [98] and may be worth exploring in cardiomyocytes. FlicR1 can be paired with green genetic tools or optogenetics, but has a relatively low signal amplitude. Unfortunately, the intensiometric operation of these GEVIs makes them vulnerable to motion artefacts during cardiomyocyte contraction.

### 3.6. Beyond Ca^2+^ and Voltage—Sensors for Cellular Energy (ATP/ADP) and Signaling Cascades (cAMP)

Tools to observe readily usable energy stores in cells (ATP/ADP), and diffusible second messenger signaling cascades (cAMP, cyclic guanosine monophosphate (cGMP), and others) have been developed that allow further mechanistic insight into single cell physiology and may have applications in cardiovascular medicine.

#### 3.6.1. ATP and ATP/ADP Indicators

ATP powers many biochemical reactions, including contraction. Therefore, direct observation of energy stores may advance understanding of disease entities where energy utilization is believed to be abnormal [99] or deficient [100]. Traditional measurement of ATP by biochemical methods lack single cell and temporal resolution. Early genetically-encoded energy sensors attempted to monitor changes through bioluminescence as ATP is consumed during the light-producing chemical reaction of firefly luciferase. In 1992, Bowers, Allshire, and Cobbold applied this to the single rat cardiomyocyte [101]. Although the light emission was weak, and the detector systems of the time were limited, the method was able to identify ATP consumption during the terminal decline of the cardiomyocyte in response to a metabolic poison, however, beat-to-beat changes were not demonstrated, and the potential pH sensitivity of the assay system prevented exact calibration of [ATP].

Brighter FP-based strategies were based on ion channel activity [102], or the conformational change of the ATP-potassium channel (K_ATP_) [103]. However, both ultimately limit ATP detection to the cell membrane and, thus, may alter cellular physiology in cardiomyocytes where the membrane-bound Na/K ATPase is essential for maintaining cell integrity. In 2009 direct measurement of total ATP in single cells was reported. The ATeam sensors, based on an improved CFP:YFP FRET pair and the epsilon subunit of a bacterial F_0_F_1_-ATP synthase enable total, and subcellular ATP observation in single cells (K_d_ ATP 2.1 mM, range 230%) [104]. The ATeam principle has been extended and improved, including the successful generation of a bioluminescent version BTeam (K_d_ ATP ~3 mM, range ~30%) that utilizes NanoLuc (and, therefore, consumes oxygen during the light-emitting reaction rather than ATP, like firefly luciferase), and Venus to produce a ratiometric signal of adequate brightness and intensity to be seen in single HeLa cells [105]. Extending the experience from the Ca^2+^ indicators, a single FP ratiometric excitation probe, QUEEN, has also been described with a slight improvement to the dynamic range (K_d_ ATP 4.5 mM, range 300%) [106]. To date they have not been applied to single cardiac cells.

Around the same time as the ATP sensors were produced, Perceval, a reporter of ATP:ADP ratio was described: utilizing cpVenus [107] and a PII family protein, GlnK. Perceval undergoes conformational change in response to the competitive binding of ATP and ADP to change the absorbance spectrum of cpVenus operating as an excitation ratiometric indicator (affinity for ATP, 0.04 μM, range 40%) [108]. Subsequently PercevalHR was identified following saturation mutagenesis around the ATP binding pocket, improving fluorescence response (range 800%), enabling single-cell studies of nutrient deprivation, and activity-dependent changes of ATP:ADP in cultured neuronal models [109]. The mechanism of the Perceval series relies on saturating concentrations of ATP/ADP to invoke a shift in the excitation wavelength. Therefore, the result obtained depends on the affinity for both ATP and ADP. Since the original Perceval had a ca. five-fold greater affinity for ATP, limitations in certain cell types was anticipated [108]. In particular, the cardiomyocyte has an estimated [ATP] 10 mM, [ADP] 60–100 μM, with an ATP:ADP ~100–150. PercevalHR extends the range of suitable ATP:ADP ratios, and since cardiac disease states tend to increase [ADP] and, thus, reduce the ATP:ADP ratio, once it has been shown to work in a relevant control population it should continue to work in disease mimicking studies. PercevalHR application to single cardiomyocyte studies of mitochondrial function, revealed an overshoot in the ATP:ADP in response to changing substrate supply from glucose to pyruvate, suggesting it may be useful in single-cell cardiomyocyte investigations [110].

#### 3.6.2. Cyclic Purine Metabolites cAMP and cGMP

Cyclic purine metabolites are important in the cardiovascular system. cAMP driven signaling cascades underlie many metabolic, electrical, structural, and transcriptional responses. In 2000 a CFP:YFP FRET-based cAMP sensor designed from a key effector of cAMP: protein kinase A (C-S65T) and the RII-β subunit was reported (K_d_ not stated, range 5%) [111] and subsequently applied to isolated cardiomyocytes highlighting the possibility of compartmentalization of intracellular signaling down to the single micron space scale [112]. Until recently cAMP visualization, like voltage measurement, struggled with poor indicator performance and low dynamic range. The brightest and best-performing cAMP indicator, EPAC^-SH126^ (K_d_ cAMP 9.5 μM, range 80%), is based on a CFP:YFP FRET principle using mTurquiose2 as the donor, and tandem cpVenus:Venus proteins as the acceptor sandwiched around part of the cAMP-binding Rap1 activating protein Epac [113]. However, as subcellular fusions, the Epac-based probes appear not to function equally in all parts of the cell leading to the development of CUTie (cAMP universal tag for imaging experiments, K_d_ cAMP 7.4 μM, range 19%) based on a CFP:YFP FRET pair where the regulatory subunit of PKA is split to house the YFP acceptor [114]. CUTie has been applied to single adult and neonatal cardiomyocytes in studies relevant to heart failure and hypertrophy. As a ratiometric indicator it only allows single compartment observation in one cell at a time, and the small effects seen may make its broad utilization difficult, particularly if the target cell population biology is extremely variable, for example, in iPSc cardiomyocytes.

Other classes of genetically-encoded indicators that may be of interest to the cardiac community include cGMP indicators, the intracellular second messenger generated by nitric oxide signaling. cGULL [115], a green intensiometric indicator based on citrine with a mouse phosphodiesterase 5α cGMP sensor domain appears to have the best single-cell characteristics (K_d_ cGMP 1 μM, range 750%) of current probes. A number of reactive oxygen species and redox indicators, summarized in [116], are described; these include the serendipitous observation that mitochondrial-targeted cpYFP flashes arising from single cardiomyocyte mitochondria, are due to bursting superoxide production. These mitoflashes have subsequently been ubiquitously detected in all eukaryotic species examined and may be part of an autoregulatory feedback that helps control ATP production in the cardiomyocyte [110].

## 4. Genetically-Encoded Actuators (Optogenetics) in Single Cardiac Cells

Optogenetic actuators are light-sensitive proteins that change their conformation and activity when stimulated by a particular wavelength of light. Since light can be controlled with sub-micrometer, and sub-millisecond precision this toolbox delivers high spatiotemporal selectivity. As the tools are genetically encoded, they can be targeted within cells by signal tags, or restricted to certain cell-types by promoter controls. They, therefore, complement the optical probes used to visualize intracellular physiology discussed above. Since they utilize the same microscopy infrastructure simultaneous experiments to control and observe responses in single cells can be delivered on a time and space scale unimaginable with other techniques.

**Figure 3:**
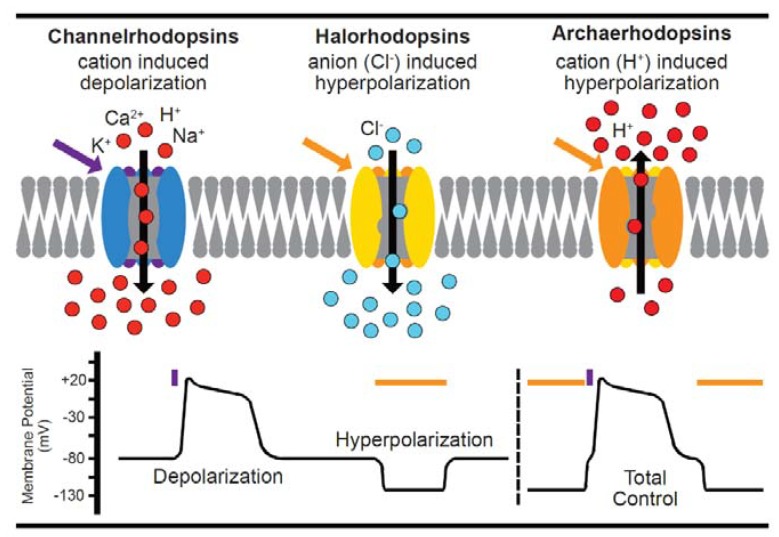
A summary of optogenetic actuators. Depolarizing channelrhodopsins, e.g., ChR2, open in response to light in the blue-green spectrum allowing positively-charged ions into the cell, raising the membrane potential and triggering the depolarization threshold. Conversely, inhibitory channels like Halorhodopsin (which pumps negatively-charged ions in) or Archaerhodopsin (which pumps protons out) hyperpolarize excitable membranes. Inhibitory channel activity is controlled by light in the green-red spectrum. The lower panel stylizes the effect on cell membrane potential by blue or orange pulses of light to depolarize, or hyperpolarize the cell. The two approaches can be combined to hyperpolarize, and then activate, the cell [95]. A number of reporter strategies discussed in this review can be integrated with stimulatory optical control [42,60,68,69,78,81,83,85,97].

Although single cell applications of optogenetic tools in other systems have been used to activate a variety of transmembrane receptor signaling pathways, activate cyclic nucleotide second messenger systems, regulate transcriptional and/or even epigenetic states [117], this extended toolbox is currently underutilized in cardiac cells. To date, cardiovascular applications have almost exclusively used optically-controlled ion channels to depolarize (e.g., Channelrhodopsin, ChR2, a class I bacterial opsin isolated from *Chlamydomonas reinhardtii* which requires light in the blue-green spectrum) the cell and thereby activate contraction. Typically, these have been undertaken in whole animals [118], explanted tissues [119], or 2D cultures [120] as the field investigates alternatives to traditional pacing, or pharmacotherapy to control heart rate or rhythm [121]. Hyperpolarizing tools (e.g., NpHR, the chloride pump Halorhodopsin isolated from *Natronomonas pharaonis* which requires light in the green-red spectrum) are also described but, to date, have not been widely used in cardiac applications. A schematic of current activating, and inactivating optogenetic tools is presented in Figure 3.

Feasibility of cardiac application of optical control in single cells owes much to the Entcheva lab: the leading early adopters of single cell control, either by viral transduction [122] or by the use of “spark cells” which electrically couple with (canine) primary cardiomyocytes, or a stem cell-derived counterpart [122,123]. A major limitation of the adult ventricular primary cell model is the limited in vitro viability of the cells (typically <24 h). This precludes the use of transient transfection/transduction systems as the rising dawn of gene expression must battle with fading cell viability.

The tandem cell unit strategy overcomes this [123] as the transgene (in this case ChR2) is stably expressed in an immortal cell line which can be co-cultured with cardiomyocytes to allow coupling between the two cell types. Alternative cell autonomous strategies for primary ventricular cardiomyocytes include transgenic ChR2 mice [124], in vivo myocardial injection of adenoviral expression systems with cardiomyocyte isolation after a week for mice [68], or in vitro infection for the relatively long-lived vGPCM [68]. vGPCM is perhaps the best rodent model of the physiological system in humans, demonstrating in vitro viability up to 72 h in our hands. A lentiviral gene delivery strategy of a more light-sensitive, and more divalent cation-selective ChR2 variant [125], has been demonstrated in the neonatal atrial cardiomyocyte model [126].

In summary, triggered depolarization of virtually all contemporary biological models of the heart/cardiomyocyte can be successfully achieved using a variety of gene delivery methods, and a number of ChR2 variants. Studies comparing the complete spectrum of ChR2 variants in particular cardiomyocyte models are lacking, therefore, currently there is no dominant strategy for a given system. At the single cell level, optical pacing has been demonstrated in iPSc/ESC cardiomyocytes, neonatal cardiomyocytes, and adult cardiomyocytes.

Like any toolbox with a decade of enabling research there are inevitable pitfalls for those wishing to adopt these approaches without guidance. Since the greatest immediate application of optogenetics in cardiac research appears to be the capability to replace traditional electrical stimulation, we will focus on the considerations relevant to excitatory (AP generating) tools, the prototype for which is ChR2. ChR2 turns on (<5 ms) and off (<50 ms) quickly enough [127] to make it a suitable tool for cardiomyocyte stimulation at frequencies up to 10 Hz. Subsequent protein engineering efforts have improved key parameters, including light sensitivity, and speeds of activation and inactivation, in addition to the magnitude and ion selectivity of the currents evoked [68]. Community resources to catalogue developments and guide the researcher have been proactively developed in this field, e.g., openoptogenetics.org or the optogenetics resource center maintained by the Deisseroth lab. To ensure that as projects are devised/funded the most appropriate tool is utilized and any caveats anticipated before peer review exposes errors, periodic attention to these repositories is encouraged. A Twitter bot for community questions (@Optoquestion) has also been established.

### Considerations for Optogenetic Application in Cardiomyocytes

Successful optical stimulation requires a light-induced current large enough to trigger depolarization. This depends on many experimental variables: gene expression, power density, and wavelength of stimulating light energy, and in the case of syncytial approaches, the magnitude of conductance between the cell expressing the optical tool and the cardiomyocyte target. Considerations for the successful application of ChR2-based stimulation include:

**Gene expression:** Since transfection of ventricular or stem cell-derived cardiomyocytes is generally a thankless task, viral delivery systems are needed. Where control and phenotyping tools are desired, the compact size of ChR2 (737 amino acids, 77 kDa) means it can be expressed with a 2A peptide and a phenotyping tool, particularly if the typical YFP fusion used to follow ChR2 infection and membrane localization is replaced by a small peptide tag, like the myc epitope [68]. Since gene expression, and auto-catalytic 2A peptide cleavage take time, we usually do not try to run an experiment until 48 h post infection. Unfortunately, the more mature the cardiomyocyte is the harder it is to infect. Typically, we need multiplicities of infection four orders of magnitude more for adult vGPCM compared to iPS-CMs [30]. Transient transfection typically generates low μM expression levels [60]. By contrast knock-in approaches with endogenous promoters may be three orders of magnitude lower. This may ultimately prove insufficient or run the risk of altering cell behavior due to insertional mutagenesis. Stable long lived RNA’s of ChR2 are commercialized by Ncardia as an alternative to viral delivery for iPSc cardiomyocytes to circumvent this issue.

**Excitation (light sensitivity):** ChR2 has a broad excitation spectrum that extends from UV to green parts of the visible spectrum [121], with optimal stimulation at 480 nm. Generally, this forces the use of phenotyping tools with excitation/emission into the red end of the visible spectrum to prevent inadvertent activation of ChR2 (see *cross talk*, below). The power density of stimulating light is an important, and often ignored, parameter. In simple terms low gene expression can be offset by application of more light (either by higher power, longer duration, or wider area) to boost the probability that sufficient channels will open and depolarize the cell. Full activation of ChR2 requires 10^18^–10^19^ photons/s/cm^2^ at 480 nm [125]. Typical power densities for single cell application in the range of 0.1 to 10 mW/mm^2^ 10 ms, which can be generated by most light-sources equipped on standard microscopes. The choice of light source should depend on the needs of the user rather than what is available. For example, the advantage of coherent light sources (lasers) is that confocal platforms are designed to deliver these with subcellular precision so individual cells can be targeted within a field, or even regions within a single cell [68], but lasers produce higher power than LED sources. This can reduce viability and experimental duration, particularly if confocal imaging is employed during phenotyping, as this is both high power and slow. Our solution to this was to develop a microscope using the lower power of LED light combined with the high sensitivity and speed of the electron-multiplying charge-coupled device (EMCCD) camera to provide continuous imaging while a laser power source can operate independently to control the cell on millisecond and subcellular space scales [68].

Alternative solutions to the problem are described; for example, the EMCCD chip architecture limits the speed of imaging due to the dead-time needed to discharge information from the detector. Since this is longer (~40 ms) than the time needed to stimulate the optical control tool, a very elegant solution from the Nagai lab has been to hide the optical stimulation pulse inside the camera dead-time [128] so that imaging is not interrupted by stimulation at all.

The dependence on relatively high light power requirements for ChR2 activation can be exploited. As outlined above, the power-density of bioluminescent probe emissions is low, typically >5 orders of magnitude (~nW/mm^2^ range) less than that needed to activate ChR2. This means that bioluminescent probes cannot inadvertently activate the optical control tool even though they may occupy the same part of the visible spectrum. As bioluminescence eliminates the need for extrinsic light for imaging, and by virtue of low-power light emission liberating the full range of the visible spectrum for phenotyping tools, they should become increasingly preferred as the brightness of bioluminescent tools improves allowing production of calcium [87,88,89,129] and voltage indicators [95]. However, caution may be required if the tandem cell unit strategy is used for activation as minimal irradiance needed in that system was 6 μW/mm^2^ [123].

**Crosstalk:** Crosstalk can be minimized by the choice of reporter, reduction of light power intensity, or spatial restriction of excitation light in multicellular applications. Indicator strategies compatible with ChR2 are highlighted within this article [42,60,68,69,78,81,83,85,97]; but combining optical stimulation with imaging probes or proteins needs a little more consideration than multi-color imaging for two reasons. Firstly, ChR2 has a broad excitation spectrum of 400–520 nm (peak 480 nm). In general, it is recommended to choose indicators with an excitation spectrum as far from ChR2 as possible to avoid inadvertent activation of the cell during dynamic imaging. If this happens the measured signal, the intensity will drift upwards [130], whereas typically slow dye photobleaching should be expected to cause signal baseline intensity to drift downwards. Secondly, the user must be conscious of the potential for the light used for ChR2 stimulation to cause photoactivation or photoconversion of the reporter probe. Many of the current genetically encoded red indicators that give rise to the RCAMP’s hail from proteins with complex excitation spectra with chromophore maturation through non-red intermediates. Residual properties of the parental protein can be retained in its progeny, but only unmasked by application of high energy blue:green light. This complicates the application of these tools with optogenetics. Furthermore, many of the leading fluorescent GEVI’s appear to have undesirable properties when combined with optical control strategies [95].

**Coupling:** When mixed cell preparations are used, conductance for successful depolarization between the ChR2-expressing activating cell and the cardiomyocyte must exceed a minimum value of about 2 nS, similar to that when the activating cell expresses HCN2, the channel that normally produces the physiological I*f* current underlying biological pacing [131]. Since coupling depends on the physical contacts established between two cell types it will be influenced by the methods to isolate and singularize cells (particularly the extracellular proteases). This process may be additionally vulnerable to how cells are handled following isolation, for example, shaking, or differential rates of sedimentation of large cardiomyocytes compared to smaller spark cells. Fortunately, as depolarization is an all-or-none phenomenon this approach appears not to be exquisitely sensitive to the ratio of cell types present.

**Cofactors:** The active chromophore in ChR2 is all-trans-retinal which is produced from vitamin A. In cultured cells it is expected that this co-factor may become limiting, encouraging some to supplement media accordingly [132]. This may not be without biological consequence for stem cell-derived models in particular.

**Ion selectivity:** ChR2 exhibits inward monovalent cation selectivity (H^+^ > Na^+^ > K^+^ > Ca^2+^) with minimal outward current. Its reversal potential is close to 0 mV. Single-channel conductance estimates have been in the 0.04–2 pS range [133] approximately one tenth (or less) of the sodium channel. This may make the tool unsuited to traditional single cardiomyocyte experiments that are conducted in Na^+^-free conditions.

**Voltage sensitivity:** Although not widely reported ChR2 has a voltage dependent activation and inactivation. At more negative potentials (−80 mV), currents are 10 times higher than at −20 mV [123]. This factor can become limiting in iPSc cardiomyocyte populations where historical methods of differentiation generate cells with resting membrane potentials in a range from −40 mV to −60 mV [134] perhaps due to the relative lack of dominant rectifier current in humans (I*_K1_*). This, therefore, will make certain cells within iPSc cardiomyocyte differentiations harder to excite than others.

**Stimulation train:** ChR2 can exist in a lower conductance, light-adapted, state meaning that chronic fast optical pacing trains may cause partial inactivation of ChR2. Experimentally, 100 s of 2 Hz stimulation using 10 ms of 480 nm light in ventricular myocytes leads to a 22% reduction in current [134].

**Heat:** ChR2 conductance can be affected by temperature where, in general, higher temperatures lead to larger currents [127]. Although the energy in electrical pacing may heat the perfusion media; no such practical concerns are apparent for light-based stimulation where typical irradiances in the low mW/mm^2^ range are theoretically predicted to have a minimal temperature change (0.013 °C for a 5.5 mW/mm^2^ pulse for 90 ms) [135].

## 5. Conclusions

Single-cell cardiomyocyte studies are essential. Protein-based tools now offer the ability to control and observe various aspects of the inner working of living cells at video rates. Naturally-occurring bioluminescent tools launched this field, and in the intervening years since they were first applied, significant knowledge and capability have developed to improve the natural offerings for bespoke disease relevant applications. To date the current tool set is underutilized in cardiovascular medicine, but we hope the description of probes available, and the infrastructure needed to use them, in this article will help encourage their transition into the space currently dominated by chemical dyes which have historical, and contemporary, evidence of toxicity in the cardiomyocyte. In the short term it would appear that most of the improvements in probe development will be directed towards neurobiological application, but it appears possible that not all of these advantages will translate to successful cardiac application.

## Figures and Tables

**Table 1:** A summary of structures, (in vitro) *K*_d_ and excitation/emission wavelengths of commonly used chemical indicators to investigate cardiac calcium handling.

**Indicator**	**Structure**	**Ca ^2+^ K_d_ (nM)**	**Excitation/Emission**	**Reference**
BAPTA	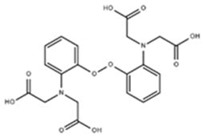	160	Non-fluorescent	[2]
FLUO-3	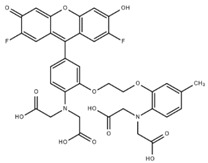	325	Ex 506 nmc Em 526 nm	[6]
FLUO-4	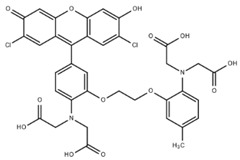	345	Ex 494 nm Em 516 nm	[7]
**Indicator**	**Structure**	**Ca^2+^ K_d_ (nM)**	**Excitation/Emission**	**Reference**
FLUO-5-N	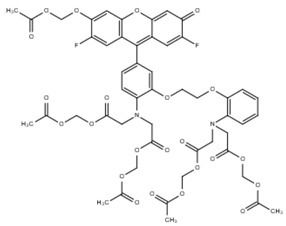	135 × 10^3^	Ex 491 nm Em 516 nm	[8]
FURA-2	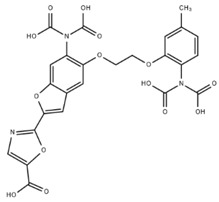	145	Ex 340/380 nm Em 510 nm	[9]
INDO-1	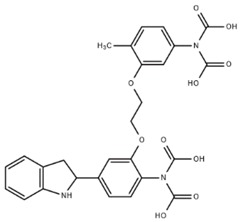	230	Ex 388 nm Em 400/475 nm	[9]
QUIN-2	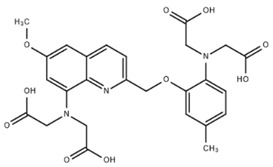	115	Ex 332 nm Em 493 nm	[3]
RHOD-2	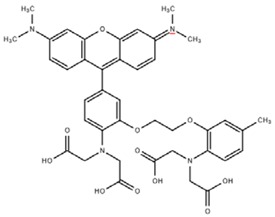	370	Ex 553 nm Em 576 nm	[6]

**Table 2:** A summary of structures and excitation:emission wavelengths of commonly used chemical indicators to investigate cardiac deplolarization.

**Indicator**	**Structure**	**Excitation:Emission**	**Reference**
SBFI (Na^+^)	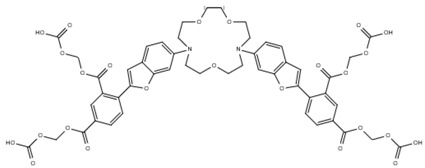	Ex 340/380 nm Em 510 nm	[31]
di-4-ANNEPS (voltage)	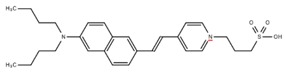	Ex 496 nm Em 705 nm	[34]
di-4-AN (F)EPPTEA (voltage)	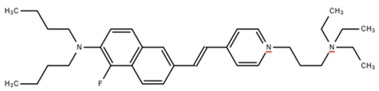	Ex 444 nm Em 610 nm	[36]
di-4-AN BDQPQ (voltage)	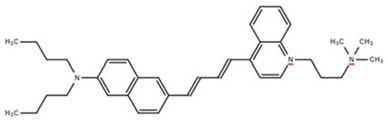	Ex 488 nm Em 680 nm	[37]
FLUOVOLT (voltage) VF2.4.Cl	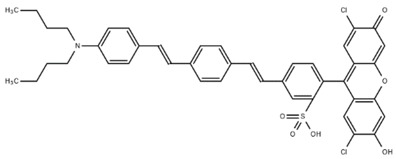	Ex 520 nm Em 551 nm	[35]
